# Long-term efficacy and safety of fractional 1064-nm picosecond laser for minimization of post-mammoplasty scar in Asians: a prospective randomized controlled study

**DOI:** 10.1007/s00403-025-04280-1

**Published:** 2025-05-28

**Authors:** Panwadee Thongjaroensirikul, Rona Maria R. Abad-Constantino, Supisara Wongdama, Visnu Lohsiriwat, Woraphong Manuskiatti

**Affiliations:** 1 Department of Dermatology, Faculty of Medicine Siriraj Hospital, Mahidol University, 2 Wanglang Road, Bangkok, 10700 Thailand; 2 Department of Surgery, Faculty of Medicine Siriraj Hospital, Mahidol University, Bangkok, Thailand

**Keywords:** Picosecond laser, Fractional 1064-nm picosecond laser, Surgical Scar, Scar minimization, Scar mitigation

## Abstract

Various types of lasers and energy-based devices have been used to prevent scar formation following primary surgical wound closure. While picosecond lasers have demonstrated potential in treating hypertrophic scars, limited data exists regarding their role in minimizing surgical scar formation. This study aimed to evaluate the safety and effectiveness of a fractional 1064-nm picosecond laser in reducing scar formation after mammoplasty in Asian patients. Eighteen patients with bilateral mammoplasty scars were enrolled, and treatment commenced within one month after surgery. For each patient, one side was randomly selected to receive four laser treatment sessions at four-week intervals, while the contralateral side served as an untreated control. Scar width and thickness were measured using a calibrated caliper, and additional evaluations were performed using the Antera^®^ 3D imaging system and the Patient and Observer Scar Assessment Scale (POSAS). Seventeen patients completed the study. Significant reductions in scar width and thickness were observed on the treated side, with average reductions of 75.3% and 84.9%, respectively, six months post-treatment. In contrast, no significant changes were found in the untreated scars. Imaging and subjective assessments further supported the efficacy of the laser treatment. The procedure was well-tolerated, with only mild and transient postinflammatory hyperpigmentation (PIH) reported in 11% of patients. These findings support the fractional 1064-nm picosecond laser as a safe and effective prophylactic option for minimizing postsurgical scarring in Asian patients.

## Introduction

Postsurgical scars pose a complex dermatological challenge, often resulting in significant physical and psychological impacts. Laser and energy-based device therapies, including the 532-nm potassium-titanyl-phosphate (KTP) laser, pulsed dye laser (PDL), fractional CO_2_ laser, fractional 1550-nm Er: Glass laser, fractional 2940-nm Er: YAG laser, light-emitting diode (LED), and intense pulsed light (IPL), have emerged as transformative approaches for scar reduction and aesthetic improvement [1–3]. Recent systematic reviews and network meta-analyses highlight the beneficial effects of laser therapy in preventing surgical scar formation, especially when treatment begins as early as one week [3] to one month post-operation [2].

The potential of picosecond lasers for dermatological applications, particularly in scar treatment, has been recognized across various clinical settings. Recent studies have documented significant improvement in hypertrophic and traumatic scars following treatment with fractional 1064-nm picosecond Neodymium-Doped Yttrium Aluminium Garnet (Nd: YAG) lasers, with minimal adverse effects [4, 5]. The effectiveness of picosecond lasers is based on laser-induced optical breakdown (LIOB), which allows for non-thermal, targeted photomechanical disruption within tissues. This mechanism promotes localized collagen remodeling and tissue repair, thereby reducing scar formation while minimizing thermal injury to surrounding tissues [5]. This study aimed to objectively and subjectively evaluate the long-term efficacy and safety of fractional 1064-nm picosecond laser in minimizing postsurgical scarring.

## Methods

This prospective randomized controlled study was approved by the Siriraj Hospital Institutional Review Board granted ethical approval (Si 913/2020) and was registered at Thaiclinicaltrials.org online registry (TCTR20210730001). Written informed consent was obtained from all participants after a thorough briefing on the study details and possible implications.

### Participant selection

A total of 18 Thai participants aged 18 to 60 and with Fitzpatrick skin types (FSTs) III to V, presenting with bilateral post-mammoplasty scars of less than one month’s duration, were enrolled based on defined inclusion criteria. Exclusion criteria were rigorous, disqualifying individuals who had received any scar treatments within six months prior, those with skin infections or a history of herpes simplex virus, immunocompromised subjects, and pregnant women.

### Intervention

The intervention involved four monthly sessions using a 1064-nm picosecond laser (Discovery PICO; Quanta System), coupled with a microlens array (MLA). Treatment was randomly assigned to one side of the scar using a computerized block randomization method (block size of four). Topical anesthesia, a mixture of 2.5% lidocaine and 2.5% prilocaine cream (EMLA; AstraZeneca LP) was administered before laser application. The device settings were a fixed pulse duration of 750 picoseconds, an 8 mm spot size, a fluence of 0.5 J/cm², and a 10 Hz pulse repetition rate. Each session included two passes until a minimal degree of pinpoint bleeding was reached, signaling the treatment endpoint. Post-treatment, petrolatum ointment was applied four times daily for 1 week. Participants were instructed to avoid the use of any topical or procedural treatments for the scar throughout the study period.

### Assessment

Follow-up assessments were conducted at 1, 3, and 6 months post-final laser session. Objective scar dimensions were measured using a calibrated caliper, and the Antera^®^ 3D system was employed for detailed analysis of skin texture, scar elevation, melanin index, and erythema index. Serial photographs of the scars were obtained at baseline and every visit. Blinded dermatological evaluations using the Patient and Observer Scar Assessment Scale (POSAS) [6] were performed at baseline, after each laser session, and during follow-ups. Furthermore, patients rated their pain level during treatment for each side using a visual analogue scale, ranging from 0 (no pain) to 10 (extreme pain). Adverse events and complications were meticulously recorded at each visit.

### Statistical analysis

Changes in scar dimensions were statistically analyzed within and between groups using Repeated Measures ANOVA, identifying significant temporal variances in scar characteristics. Significant ANOVA findings led to subsequent Bonferroni’s multiple comparison tests to mitigate the risk of Type I errors. Direct comparisons of scar width and height between treatment and control sides at individual time points were made using the paired *t*-test, with significance determined at a *P*‐value of less than 0.05.

## Results

Of 18 enrolled participants, 17 completed the study protocol. Withdrawal occurred for one individual due to official obligations necessitating travel outside the province. Participant demographics are reported in Table 1.

**Table 1:** Participant demographics

Characteristics (*n* = 18)		
**Age, (years; mean ± S.D.)** (min to max)		37.9 ± 5.64 (29–48)
**Sex, n (%)**	Male	0 (0%)
	Female	18 (100%)
**Skin type, n (%)**	III	5 (27.8%)
	IV	12 (66.7%)
	V	1 (5.6%)
**Breast surgery indicated for,** n (%)	Breast cancer	3 (16.7%)
	Breast augmentation	10 (55.6%)
	Breast reduction	5 (27.8%)
**Age of scar at initiation of prophylactic treatment,** (days, mean ± S.D.) (min to max)		23.78 ± 6.48 (15–31)

### Caliper measurement

**Fig. 1:**
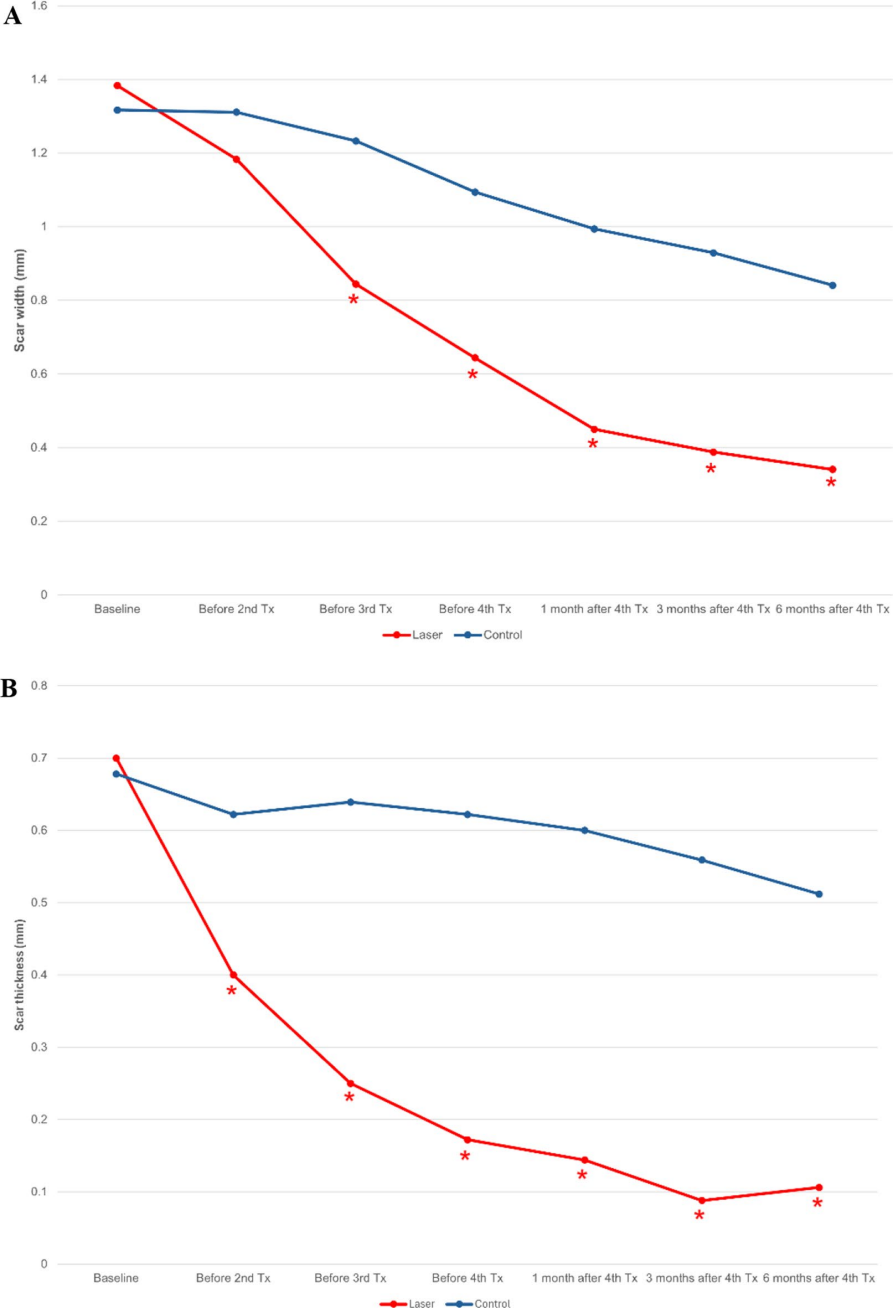
Average scar width (**A**) and scar thickness (**B**) measured using a caliper. * indicates a significant difference between interventions. mm stands for millimeters. Tx represents treatment

**Fig. 2:**
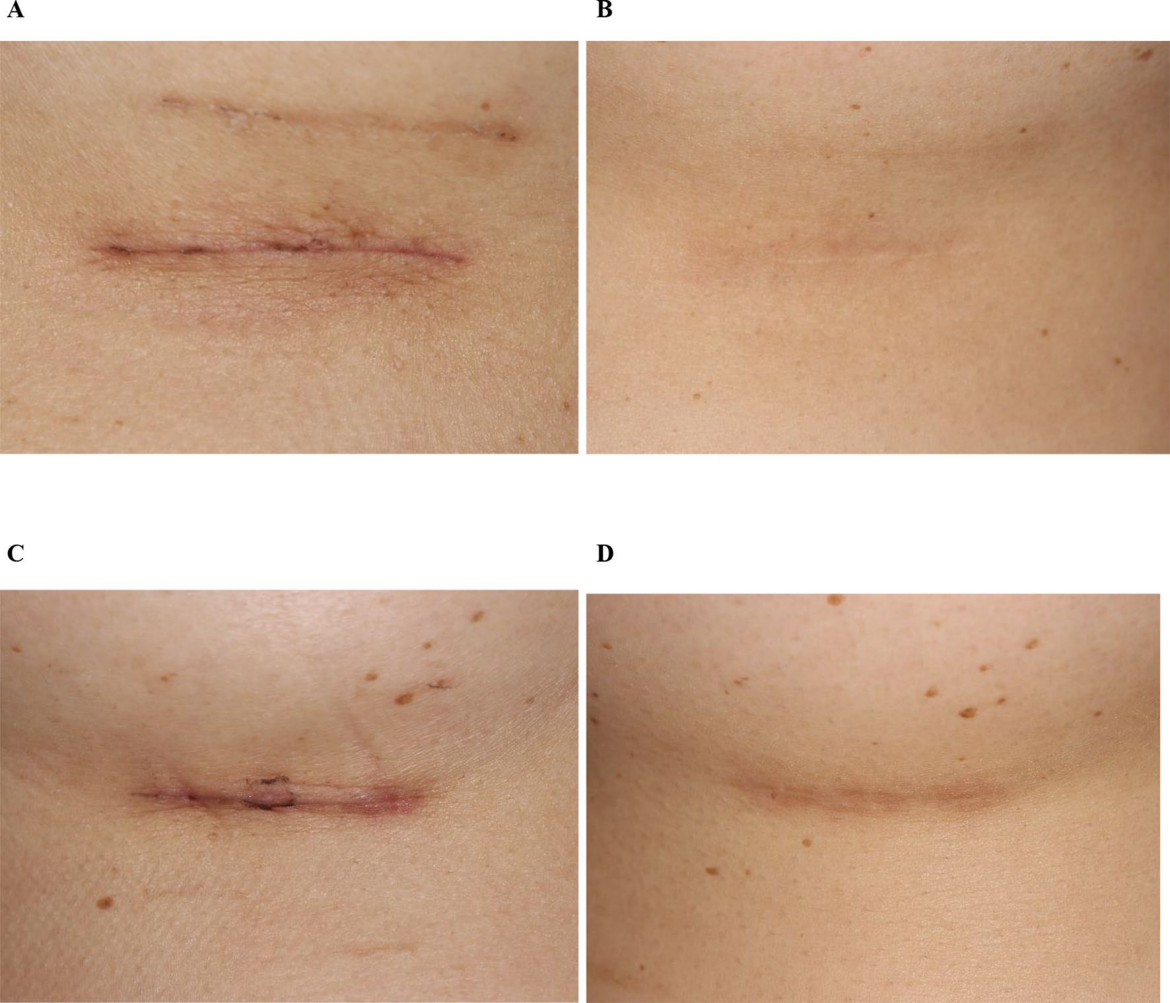
Clinical progression of post-mammoplasty scars in a 37-year-old subject with Fitzpatrick Skin Type III. (**A**) Baseline image of a 3-week-old scar on the treatment side; (**B**) 6 months after four treatment sessions. (**C**) Baseline image of a 3-week-old scar on the untreated control side; (**D**) scar appearance 11 months after no treatment

**Fig. 3:**
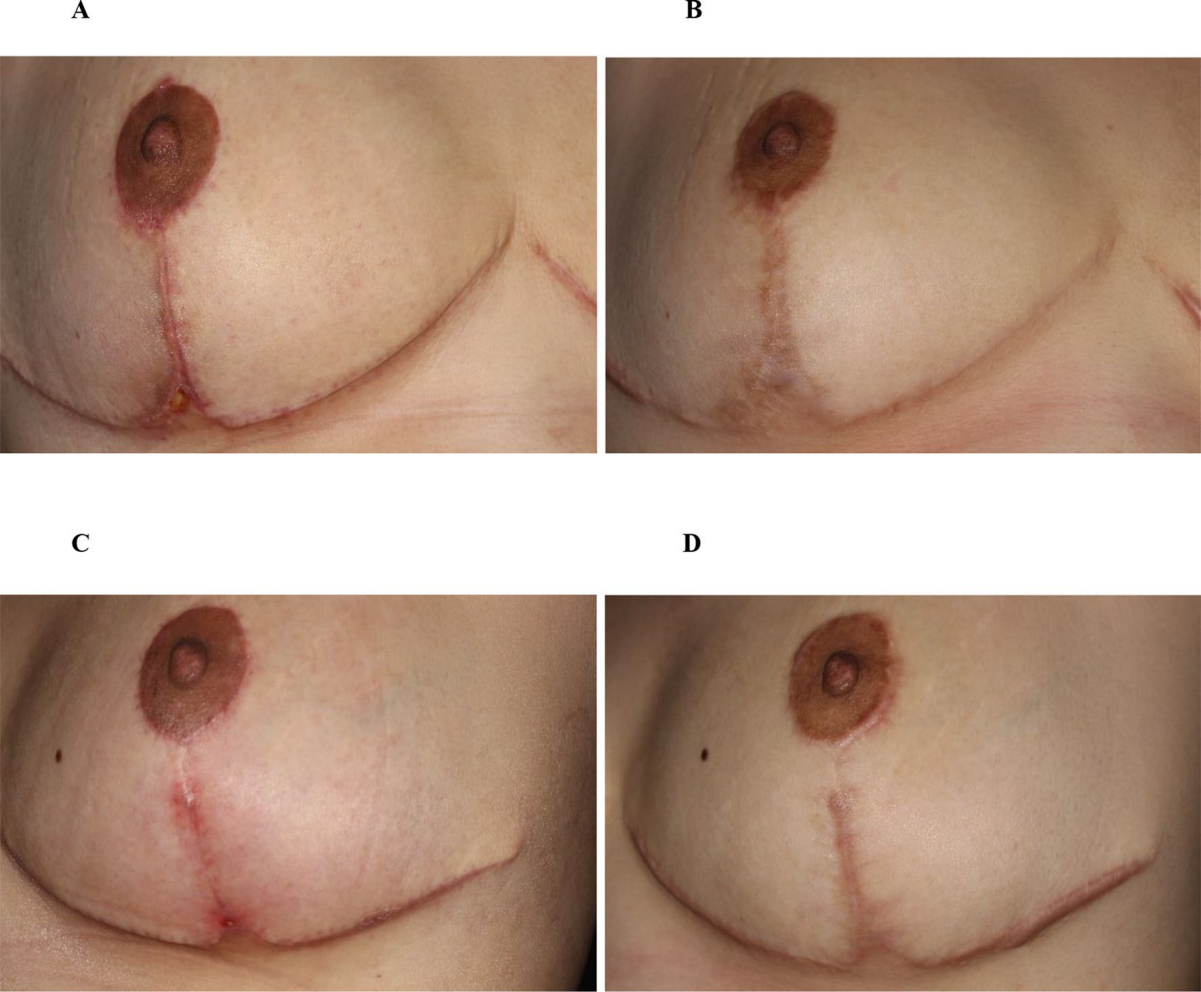
Clinical progression of post-mammoplasty scars in a 35-year-old subject with Fitzpatrick Skin Type IV. (**A**) Baseline image of a one-month-old scar on the treatment side; (**B**) 6 months after the final (4th) treatment. (**C**) Baseline image of a one-month-old scar on the untreated control side; (**D**) scar appearance 11 months after no treatment

After four monthly treatments, there were significant reductions in both the average width (*p* < 0.001) and thickness (*p* < 0.001) of scars on the treatment side compared to baseline (Fig. 1). The average scar width on the treatment side decreased from 1.383 ± 0.970 mm (mean ± S.D.) at baseline to 0.341 ± 0.283 mm at six months post-final (4th) treatment, representing an average reduction of 75.3%. Similarly, the average scar thickness decreased from 0.700 ± 0.283 mm at baseline to 0.106 ± 0.178 mm at six months post-final treatment, resulting in an average reduction of 84.9%. In contrast, there were no significant changes in the width (*p* = 0.063) and thickness (*p* = 0.063) of scars in the untreated control group across all follow-up visits (Fig. 1). Figures 2 and 3 illustrate the clinical appearance of the scars on both the treatment and untreated control sides at baseline and at the 6-month follow-up visits for two patients.

### Antera 3D imaging system assessment

The scar texture on the treatment side showed significant improvement throughout the 6-month follow-up period (*p* < 0.001), with an average improvement of 29.4% in scar texture values (from 13.465 ± 4.835 at baseline to 9.512 ± 3.962 at the final follow-up visit). A significant improvement in scar texture on the treatment side was observed as early as 1 month after the third laser treatment (*p* = 0.024). Interestingly, the scar texture on the control side also exhibited significant improvement during the 6-month follow-up (*p* = 0.047), with an average improvement of 25.4% in texture values (from 12.120 ± 2.857 at baseline to 9.040 ± 2.387 at the final follow-up visit). However, the significant improvement on the control side occurred much later, only becoming apparent 10 months after remaining untreated.

Scar elevation on the treatment side significantly decreased from baseline to the final follow-up visit (*p* = 0.003), with an average reduction of 44% in scar elevation values (from 0.634 ± 0.354 at baseline to 0.355 ± 0.343 at the 6-month follow-up). In contrast, the control side showed no significant reduction in scar elevation from baseline to the last follow-up visit (*p* = 0.137).

The average melanin level of the scars on the treatment side significantly decreased from 0.691 ± 0.086 to 0.653 ± 0.109 over the 6-month period following the final treatment (*p* = 0.027). In contrast, melanin levels on the control side did not show significant changes from baseline to the last follow-up visit (*p* = 0.056). An average reduction of 5.5% in melanin levels was observed 6 months after the last treatment. The significant reduction in melanin levels on the treatment side was first noted at the 3-month follow-up (*p* = 0.038) and remained consistent throughout the final follow-up.

The average hemoglobin (erythematous) level of the scars on the treatment side significantly decreased from a baseline value of 1.443 ± 0.337 to 1.154 ± 0.252 at the 6-month follow-up (*p* < 0.001). Similarly, the average erythematous level on the control side also showed a significant reduction, decreasing from 1.485 ± 0.304 at baseline to 1.228 ± 0.278 at the 6-month follow-up (*p* = 0.002). Notably, significant reductions in hemoglobin levels were observed earlier on the treatment side, as early as 1 month after the second treatment (*p* = 0.016), compared to the control side, where significant reductions were only seen 6 months after the final treatment session (*p* = 0.047).

### Patient and observer Scar assessment scale

**Fig. 4:**
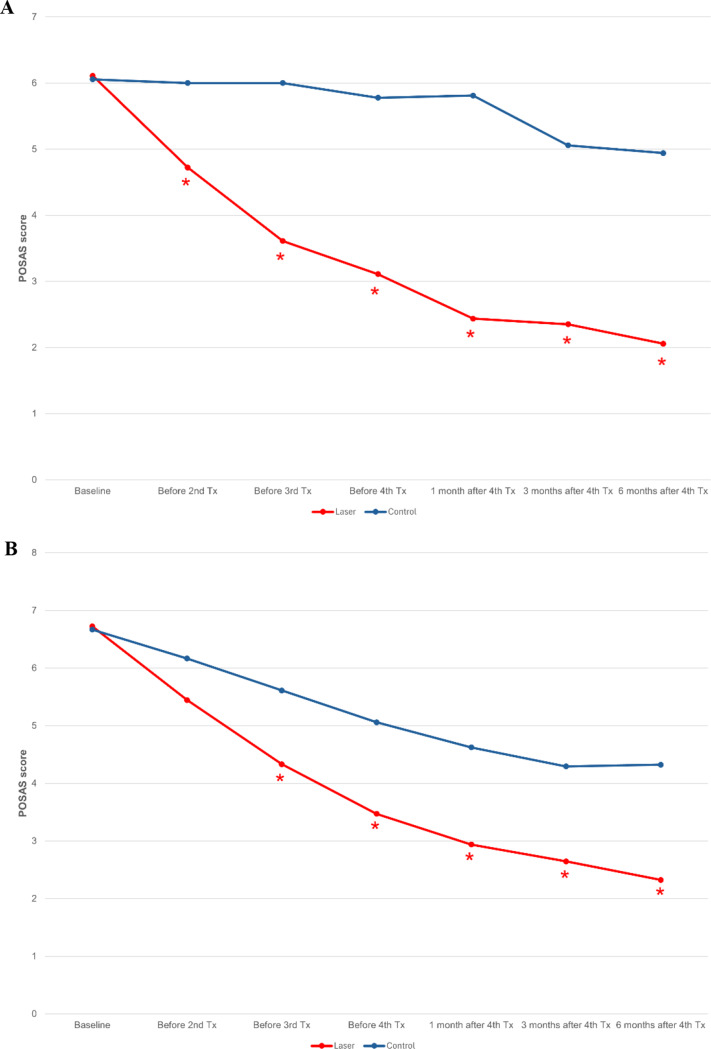
Patient and Observer Scar Assessment Scale (POSAS). (**A**) Observer Scar Assessment Scale, (**B**) Patient Scar Assessment Scale. * indicates a significant difference between interventions. Tx denotes treatment

Prior to treatment, the observer portion of the POSAS scores showed no significant difference between the treatment and control sides (*p* = 0.749). Eventually, the mean observer POSAS scores in the treatment (*p* < 0.001) and the control sides (*p* = 0.003) showed significant improvement compared with baseline (Fig. 4A). In the treatment side, a significant improvement from baseline was noted as early as 1 month after the 1st treatment (*p* = < 0.001), whereas in the control side a significant improvement from baseline was initially reported at 10 months (*p* = 0.013). The mean of observer POSAS scores in the treatment side were significantly lower than the scores in the control sides starting at 1 month after the first treatment and at all follow-up visits.

At baseline, the patient portion of the POSAS scores also showed no significant difference between the treatment and control sides (*p* > 0.999). By the end of the study, the mean POSAS scores in treatment (*p* < 0.001) and control (*p* < 0.001) sides showed significant improvement compared with baseline (Fig. 4B). Significant improvement of patient POSAS scores in both the treatment and control sides were reported at 1 month after the first treatment and were improved at all follow-up visits. Similar to the observer POSAS scores, the patient POSAS scores in the treatment side were significantly lower than the scores in the control side. This trend was observed starting after the second treatments (*p* = 0.005) and was maintained at all subsequent follow-up visits (*p* < 0.001, = 0.002, 0.007 and 0.005 at 1 month after the third and at 1, 3 and 6 months following the fourth treatment, respectively).

### Adverse effects

Mild erythema, which resolved within an average of 3.29 ± 2.24 days, was observed in all patients. Additionally, tiny grid-like scabs lasting an average of 2.20 ± 0.84 days were noted in all subjects. Mild post-inflammatory hyperpigmentation (PIH), which completely disappeared within an average of 3 ± 1.34 weeks, was documented in 11% (2 of 18) of patients. No other significant adverse effects were reported.

## Discussion

Aberrant wound healing can lead to hypertrophic or keloid scarring, which may cause functional impairment and psychological distress, particularly when the scars are located on visible areas of the body [7]. Thus, effective interventions to minimize postoperative and post-traumatic scar formation are crucial. Lasers and energy-based devices (EBDs) have become widely accepted as preventive measures against scarring [1–3]. The present study confirms the beneficial effects of early intervention with a fractional picosecond 1064-nm laser on scar morphology following mammoplasty.

While the untreated control group showed some natural improvement over time, the laser-treated scars demonstrated significantly better appearance compared to the untreated areas throughout the study period. Objective evaluations, including measurements of scar width, thickness, texture, elevation, melanin, and erythematous levels, indicated that despite the self-improvement observed in untreated scars, the treated cohort experienced more pronounced enhancements. These outcomes likely reflect the efficacy of the fractional 1064-nm picosecond laser in remodeling collagen structure and reducing excess scar pigmentation and erythematous components. The improvements in hyperpigmentation and erythema can be attributed to the ability of melanin and hemoglobin in the scars to selectively absorb light at the 1064-nm wavelength [8].

Consistent with previous studies [9, 10], the present trial observed substantial self-improvement in the vascularity (erythematous element) of untreated post-mammoplasty scars over time. Significant improvement in the color of untreated scars was noted by the 6-month follow-up, when the scars were nearly one year old. In contrast, the treated group showed improvements at earlier follow-up visits, suggesting that picosecond laser treatment may accelerate the resolution of the erythematous component in immature scars.

The fractional 1064-nm picosecond laser has also been demonstrated as effective for treating mature atrophic traumatic and surgical scars without significant adverse effects [5, 11]. The energy parameters used for treating mature scars are more aggressive compared to those employed for scar minimization following primary surgical closure in this study. Disphanurat et al. [5] used three passes of the fractional 1064-nm picosecond laser with a 6- or 8-mm spot size and a fluence of 1–1.2 J/cm² for mature atrophic traumatic and surgical scars. In contrast, this study utilized only two passes with an 8-mm spot size and a fluence of 0.5 J/cm². We opted for a more conservative parameter setting to minimize the risk of adverse effects on immature scars. As a result, the adverse effects observed in this study were minimal and transient, while the intervention proved effective for scar reduction.

While objective assessments, including caliper measurements and Antera^®^ 3D imaging system analyses, highlighted the benefits of fractional 1064-nm picosecond laser treatment compared to untreated sides of post-mammoplasty scars, the subjective evaluation using POSAS scoring also indicated improvements. While the observer and patient portions of the POSAS scores for the untreated scars did show improvement at later follow-up visits, the laser-treated scars demonstrated significantly better outcomes. The POSAS scores for the laser-treated scars consistently remained superior to those of the untreated scars at nearly all follow-up visits.

A recent systematic review and network meta-analysis indicated that low level laser therapy (LLLT) [12] and pulsed dye laser (PDL) [13] are among the most effective treatments for minimizing scar formation following primary closure of surgical wounds, with comparable treatment outcomes [3]. However, the fractional 1064-nm picosecond laser, while effective, has some drawbacks compared to LLLT and PDL. Notably, the fractional 1064-nm picosecond laser is associated with treatment-related pain and a wounding appearance, whereas LLLT and PDL are considered nonablative laser systems with fewer such side effects. Despite this, the fractional 1064-nm picosecond laser offers additional benefits in improving scar texture and pigmentation. These advantages and disadvantages should be thoroughly discussed during patient consultations.

Acknowledging the study’s limitations is essential. The small cohort size, while informative, suggests caution in generalizing the results. Additionally, the absence of patient blinding may have introduced subjectivity into the self-assessment scores, a factor that future research designs should address to preserve the integrity of patient-reported outcomes. Furthermore, the anatomical regions of the scars included in this study may not fully represent the responsiveness or the risk of adverse effects in other anatomical areas of the body.

In summary, this study demonstrates that the fractional picosecond 1064-nm laser is effective in minimizing post-mammoplasty scar formation in dark-skinned individuals, with minimal and transient adverse effects. Future research with larger sample sizes and double-blind methodologies is crucial to further establish the role of picosecond lasers in scar prevention and management strategies.

## Data Availability

No datasets were generated or analysed during the current study.
